# Stigma and discrimination against people living with HIV by healthcare providers, Southwest Ethiopia

**DOI:** 10.1186/1471-2458-12-522

**Published:** 2012-07-13

**Authors:** Garumma T Feyissa, Lakew Abebe, Eshetu Girma, Mirkuzie Woldie

**Affiliations:** 1 Department of Health Education and Behavioral Sciences, Jimma University, Jimma, Ethiopia; 2 Department of Health Services Management, Jimma University, Jimma, Ethiopia; 3 P.O. Box 1637, Jimma, Ethiopia

**Keywords:** Stigma and discrimination, Healthcare providers, HIV/AIDS

## Abstract

**Background:**

Stigma and discrimination against people living with human immunodeficiency virus (HIV) are obstacles in the way of effective responses to HIV. Understanding the extent of stigma / discrimination and the underlying causes is necessary for developing strategies to reduce them. This study was conducted to explore stigma and discrimination against PLHIV amongst healthcare providers in Jimma zone, Southwest Ethiopia.

**Methods:**

A cross-sectional study, employing quantitative and qualitative methods, was conducted in 18 healthcare institutions of Jimma zone, during March 14 to April 14, 2011. A total of 255 healthcare providers responded to questionnaires asking about sociodemographic characteristics, HIV knowledge, perceived institutional support and HIV-related stigma and discrimination. Factor analysis was employed to create measurement scales for stigma and factor scores were used in one way analysis of variance (ANOVA), T-tests, Pearson’s correlation and multiple linear regression analyses. Qualitative data collected using key-informant interviews and Focus Group Discussions (FGDs) were employed to triangulate with the findings from the quantitative survey.

**Results:**

Mean stigma scores (as the percentages of maximum scale scores) were: 66.4 for the extra precaution scale, 52.3 for the fear of work-related HIV transmission, 49.4 for the lack of feelings of safety, 39.0 for the value-driven stigma, 37.4 for unethical treatment of PLHIV, 34.4 for discomfort around PLHIV and 31.1 for unofficial disclosure. Testing and disclosing test results without consent, designating HIV clients and unnecessary referral to other healthcare institutions and refusal to treat clients were identified. Having in-depth HIV knowledge, the perception of institutional support, attending training on stigma and discrimination, educational level of degree or higher, high HIV case loads, the presence of ART service in the healthcare facility and claiming to be non-religious were negative predictors of stigma and discrimination as measured by the seven latent factors.

**Conclusions:**

Higher levels of stigma and discrimination against PLHIV were associated with lack of in-depth knowledge on HIV and orientation about policies against stigma and discrimination. Hence, we recommend health managers to ensure institutional support through availing of clear policies and guidelines and the provision of appropriate training on the management of HIV/AIDS.

## Background

Since the beginning of the HIV epidemic, stigma and discrimination have been identified as the major obstacles in the way of effective responses to HIV. HIV-related stigma and discrimination is a complex social process that interacts with, and reinforces, the pre-existing stigma and discrimination associated with sexuality, gender, race and poverty [1-4]. HIV/AIDS-related stigma and discrimination occur everywhere, but they may have more serious consequences in healthcare settings [5].

A disadvantage stemming from stigma goes beyond what are often understood as discriminatory actions. These can include ―the perception that they are not at risk of the disease for those who do not know their HIV status. And for PLHIV, they can include internalized stigma, lowered self esteem, depression, and changes in behavior (e.g., not using the available services) because of the fear of stigma [6,7]. It was indicated that higher perceived HIV stigma scores amongst clients with HIV were significantly and negatively correlated with the quality of life [8]. Stigma reduced participation in programmes to prevent mother-to-child transmission of HIV (PMTCT) [9-12]. It also affects the attitudes of providers who deliver HIV-related care [6,7,13-20].

Service providers in healthcare institutions are expected to provide social and psychological support to persons living with HIV (PLHIV) in order to help them cope with stress and to reduce the stigma directed against PLHIV. However, HIV/AIDS-related stigma and discrimination have been extensively documented amongst healthcare providers. There have been many reports from healthcare settings of HIV testing without consent, breaches of confidentiality, labeling, gossip, verbal harassment, differential treatment and even denial of treatment [5,11,13-25]. People who feel stigmatized by healthcare providers face problems getting tested for HIV and accessing optimal healthcare services. The fear of stigma impedes prevention efforts, including discussions of safer sex and PMTCT [5,12,19,26-34].

Effectively addressing stigma removes what still stands as a roadblock to concerted action, whether at local, community, national or global level. Efforts to reduce stigma and discrimination related to HIV/AIDS will not only help countries reach key targets for universal access and Millennium Development Goal 6, they will also protect and promote human rights, foster respect for PLHIV and other affected groups, and reduce the transmission of HIV. The reduction of the HIV/AIDS-related stigma and discrimination amongst healthcare providers will be helpful not only for the marginalized groups, PLHIV and their associates, but also for the healthcare providers themselves. Studies indicate that healthcare providers delay from accessing healthcare services because of the fear of stigma and discrimination [35-40]. Understanding the magnitude of, and causes underlying HIV-related stigma and discrimination amongst health workers is necessary for developing anti-stigma strategies and programs [35,39,41].

Nevertheless, in Ethiopia only little knowledge exists about HIV/AIDS-related stigma and discrimination amongst healthcare providers. In addition, the previous study in Ethiopia did not utilize psychometric approaches to measure the degree of HIV/AIDS-related stigma and discrimination.

In order to combat stigma and discrimination, it is important to quantify them, to understand their magnitudes, to explore their associated factors and to explore how they vary across groups, settings and cultural contexts within a country [10]. Furthermore, no single published study has adressed the issue of HIV/AIDS-related stigma and discrimination amongst healthcare providers in healthcare institutions of Jimma zone. This study was conducted to explore stigma and discrimination against PLHIV amongst healthcare providers in Jimma zone, Southwest Ethiopia.

## Methods

### Study design

A cross sectional quantitative study, supplemented by qualitative Key-informant interview and FGD, was conducted in Limmu Genet District Hospital and in 17 health centers of Jimma zone from March 14 to April 14, 2011.

### The study context

In Ethiopia, in 2009, there were estimated to be 1.2 million PLHIV, with an adult HIV prevalence of 2.4%. The HIV/AIDS epidemic in Ethiopia is generalized, with significant heterogeneity between regional states and population groups. The major mode of HIV transmission is heterosexual; accounting for 87% of all infections [42-45]. Ethiopia has laws and regulations that protect PLHIV against discrimination. These include both general non-discrimination provisions and provisions that specifically mention HIV in relation to schooling, housing, employment, healthcare etc. Mandatory HIV testing for employment is strictly prohibited in the country’s Labor law [46]. Additionally, the Civil Service Workplace HIV/AIDS Guideline of the country protects PLHIV from discrimination by employers [47]. Governmental sectors and Non-Governmental Organizations (NGO’s) have been working hard to support implementation of these laws and regulations (e.g. Ethiopian Human Rights Commission, Federal Ministry of Labor and Social Affairs, Federal Ministry of Women’s Affairs, Ethiopian Women Lawyers Association, Women’s Coalition, Women’s PLHIV network, and others). The Ethiopian Women Lawyers Association provides free legal services to PLHIV, and there are programs to reduce HIV-related stigma and discrimination, and to raise awareness amongst PLHIV concerning their rights. The promotion and protection of human rights of people infected and affected by HIV is explicitly mentioned in the Ethiopian HIV/AIDS policy [43,44].

### Participants

Jimma Administrative Zone is found in the Oromia regional state, in the southwestern part of Ethiopia. The zone town, Jimma, is located 357 km away from the capital city of Ethiopia, Addis Ababa. In the zone, there were 54 health centers and one district hospital during the data collection period. During the data collection period (from March 14 to April 14, 2011), there were 567 healthcare providers working in the 54 health centers and 74 healthcare providers working in Limmu Genet District Hospital. All the health centers were assumed to be the same with respect to the study topic, because their staffing pattern and administration structures were all typical for the Ethiopian context. Therefore, the quantitative part of the study included all healthcare providers working in Limmu Genet District Hospital (74 healthcare providers) and in the 17 randomly selected health centers (190 healthcare providers) during the study period. This gave a total of 264 healthcare providers who could be approached for the study.

On the other hand, Serbo health center, Seka health center and Limmu Genet District Hospital were selected for the key informant interview using convenience sampling method, because these centers were suitable for referential sampling methods, in case follow up information is required. The technical heads and ART coordinators of the selected healthcare facilities and the representatives of the associations of PLHIV in Kersa and Seka Chokorsa districts were selected using case intensity sampling method, because these groups were expected to provide in-depth information. A total of four FGDs (two with males and two with females) were conducted with the members of associations of PLHIV in Serbo and Seka Chokorsa towns. Six to eight individuals were selected conveniently to participate in each of the FGDs. They were selected based on their willingness, their ability to communicate and their availability. In addition, a total of six key informant interviews (four with the heads of units in healthcare facilities and two with the representatives of associations of PLHIV) were conducted.

### Study variables

The dependent variable was stigma and discrimination against PLHIV. The independent variables were socio-demographic characteristics of healthcare providers, basic and in-depth HIV knowledge of healthcare providers, training on topics related to stigma and discrimination, perceived institutional support, the presence or the absence of ART service in the healthcare institution.

### Instruments and measures

To collect the qualitative data, interview guides and FGD guides were developed in the manner that they addressed institutional support in the context of HIV/AIDS, the existence of breaches of confidentiality, differential treatment, labeling, gossips and general interactions of healthcare providers with HIV clients and the factors affecting it.

On the other hand, pre-tested self-administered questionnaires were used to collect the quantitative data. The questionnaires were translated into Amharic (the official language of Ethiopia) and Afan Oromo (a local language) and then back-translated into English to ensure semantic equivalence. The questionnaires were then pretested on the healthcare providers in the health centers found in Jimma town (not included in the study), making 5% of the study population, before the actual data collection.

The questionnaires contained socio-demographic variables (age, sex, marital status, religion, perceived religiosity, monthly income, educational status, ethnicity, and educational qualification), personal experience (HIV case load, work experience, and previous training on topics related to HIV/AIDS-related stigma and discrimination), HIV knowledge, perceived institutional support and items to measure HIV/AIDS-related stigma and discrimination. Three items were used to measure in-depth knowledge of HIV. Basic knowledge of HIV (two items) was assessed by asking participants to identify the bodily fluids which have a high enough concentration of HIV to transmit the virus. These items were adapted from previous studies [25,48].

The clarity and cultural acceptance of each of the items was tested though major revision was not required. We have reported the validity and reliability of the scales used in this study elsewhere [49]. A Cronbach's alpha of 0.70 or greater was the cut-off point to judge the internal consistency of each scale [50,51].

The stigma scores were standardized as the percentage of the maximum scale (%SM) scores to facilitate comparison. This enables future researchers to easily compare their findings with those in this study even if they make use of different number of items and/or response categories. These scores lie between 0 and 100 [52,53].

### Data collection procedure

The quantitative data were collected by nine health professionals with a qualification of first degree, after two days of training to familiarize them with the instruments. The names in the questionnaires were replaced by codes and participants were informed so that they had a record of their own codes to facilitate tracking of the completeness of their respective questionnaires. The completeness of the data was checked on site and the codes for the incomplete questionnaires were posted for the participants. Incomplete questionnaires were put in offices arranged for the purpose so that participants could complete their own questionnaires.

The qualitative data were collected by four students attending their Masters of Public Health (MPH) program and the principal investigator. The qualitative data collectors were given two days of training on the techniques of interviewing in-depth interviews and conducting FGDs. Field practice was carried out on the skills of interviewing and transcribing verbatim.

Each FGD session was run for one and half an hour to two hours while the interview sessions lasted for about thirty minutes to one hour. Key informants were revisited when additional information was needed. The ideas obtained from the key informant interviews and FGDs were summarized for the participants before closing up the interview and FGD sessions to make sure that the ideas reflect the views of the participants (member checking). The interviews and FGDs were tape-recorded, and were transcribed immediately after the respective sessions. Daily debriefing sessions were also being conducted amongst the data collectors to collect further and detailed information based on the insight gained at each step. The principal investigator, along with two students attending their MPH program, supervised the overall data collection procedure. He used a diary record of the data collection and data analysis procedures.

### Operational definitions

1. HIV case load: Number of HIV clients for whom the healthcare provider has given professional healthcare service in the last six months

· Low HIV case load: Fewer than ten HIV clients for whom the healthcare provider has given professional healthcare service in the last six months.

· High HIV case load: More than ten HIV clients for whom the healthcare provider has given professional healthcare service in the last six months [54].

2. HIV knowledge

· Have in-depth HIV knowledge: Correct responses to at least two of the three HIV in-depth knowledge questions**.**

· No in-depth HIV knowledge: Correct response to less than two HIV in-depth knowledge questions**.**

· High basic HIV knowledge: Correctly identifying all body fluids that have high enough concentration of HIV to transmit the virus**.**

· Low basic HIV knowledge: Missing one or more body fluids that have high enough concentration of HIV to transmit the virus [48].

3. Stigma and discrimination were measured by seven scales: 

· The lack of feelings of safety had 8 items measured on four-point Likert scale, higher scores indicating higher stigma and discrimination

· Discomfort around PLHIV had 5 items measured on four-point Likert scale, higher scores indicating higher stigma and discrimination.

· The fear of work-related HIV exposure had two items measured on four-point Likert scale, higher scores indicating higher stigma and discrimination.

· The value-driven stigma had seven items measured on four-point Likert scale, higher scores indicating higher stigma and discrimination.

· Unethical treatment of PLHIV had four items measured on four-point Likert scale, higher scores indicating higher stigma and discrimination.

· Extra precaution had three items measured on four-point Likert scale, higher scores indicating higher stigma and discrimination.

· Unofficial disclosure had five items measured on four-point Likert scale, higher scores indicating higher stigma and discrimination.

4. Perceived institutional support was measured with three scales:

· The perception of supply-related institutional support had five items measured on three-point Likert scale, higher scores indicating higher perception of institutional support.

· The perception of policy-related institutional support had three items measured on three-point Likert scale, higher scores indicating higher perception of institutional support.

· The perception of protocol-related institutional support had seven items measured on three-point Likert scale, higher scores indicating higher perception of institutional support.

### Data processing and analysis

The quantitative data were checked for completeness and were entered into EPI-DATA version 3.1. After double entry verification, the data were exported to SPSS version 16.0 for analysis. The data were explored using descriptive analyses to clean data entry errors. Factor scores were created and were used in the subsequent analysis. Following that, Pearson correlation coefficients were used for examining the relationship between stigma scales and continuous variables in the bivariate analysis. The methods employed in the creation of measurement scales has been described elsewhere [49]. One-way ANOVA (analysis of variance) and independent sample T-tests were used for comparing stigma scores across the categories.

Hierarchial multiple linear regression analysis was conducted to identify significant predictors of stigma and discrimination after controlling for other independent variables. Socio-demographic variables were entered into the first linear regression model while HIV knowledge and institution-related variables were entered into the second one. The variables found to be significantly associated with the dependent variable in the preceding models were entered into a final model. Since there were many explanatory variables stepwise method was employed to enter the independent variables into the first two models. Enter method was employed to enter variables into the final model. The assumptions in multiple linear regressions (linearity, normality and multicollinearity) were checked. The qualitative data were analyzed manually using thematic analysis method and were triangulated with the quantitative data.

### Ethical considerations

The research proposal was approved by the ethical clearance committee of the Public Health and Medical Sciences College of Jimma University. A permission letter was obtained from the Jimma zone health department and the respective healthcare facilities. A written informed consent was also obtained from each study participant. The right of the study participants to refuse participation or withdraw from the study at any point was respected. All the data obtained in due course were confidentially kept. The names of the respondents were replaced with codes to ensure confidentiality.

## Results

### Characteristics of the respondents

Two hundred fifty five (response rate 96.6%) healthcare providers participated in the survey, 156(61.2%) of whom were males. The majority were from the Oromo ethnic group (72.2%). One hundred eight (42.4%) were Orthodox Christians; 160 (62.7%) were nurses and 171 (65.9%) had diploma level educational qualification; 113 (44.3%) had monthly income less than or equal to 1233 Ethiopian birr (USD equivalent to 72.96) (Table 1).

**Table 1:** Sociodemographic characteristics of healthcare providers, Southwest Ethiopia, 2011(n = 255)

**Variable**	**No (%)**
Sex	
Male	156 (61.20)
Female	99 (38.80)
Age group	
20-24	96 (37.60)
25-29	103 (40.40)
30-34	27 (10.60)
35 years or more	29 (11.40)
Marital status	
Married	125 (49.00)
Single	125 (49.00)
Separated/widowed/divorced	5 (2.00)
Ethnicity	
Oromo	187 (73.30)
Amhara	34 (13.30)
Others ^1^	34 (13.30)
Religion	
Orthodox	108 (42.40)
Protestant	72 (28.20)
Muslim	67 (26.30)
Others ^2^	8 (3.10)
Perceived religiosity	
Very religious	59 (23.10)
Somewhat religious	74 (29.00)
Non-religious	122 (47.80)
Professional category	
Nurse/ midwife	160 (62.70)
Pharmacist/druggist	30 (11.80)
Medical laboratory	29 (11.40)
Health officer	27 (10.60)
Others^3^	9 (3.50)
Work experience	
Less than 5 years	186 (72.90)
5 years or more	69 (27.10)
Educational status	
Diploma and Certificate	171 (67.10)
First degree and above	84 (32.90)
Monthly income	
1233 Eth. Birr (72.96 USD) or lower	113 (44.30)
1234-2249 Eth. Birr(73.02-133.08 USD)	80 (31.40)
2550 Eth. Birr(133.14 USD) or higher	60 (23.50)

^1^Tigre, Gurage, Dawro, Walaita, Kambata, Yem and Hadiya ^2^Wakeffata and Catholic ^3^Medical doctors, radiographers and X-ray technicians.

The average age of the participants was 27.2 (SD 5.72), ranging from 20 years to 49 years and the average work experience was 4.8 (SD 6.23) years, ranging from one month to 31 years. The average monthly income was 1631.36 Birr (96.53 USD) [SD 585.06 Birr (34.62 USD)], ranging from 700.00 Birr (41.42 USD) to 4,000.00 Birr (236.69 USD).

One hundred fifty seven (61.6%) of the survey participants had never attended training on topics related to stigma and discrimination against PLHIV. One hundred fifty one (76.1%) participants were from health centers. Sixty eight (26.7%) and 99 (38.8%) of the participants had high basic HIV knowledge and in-depth HIV knowledge, respectively (Table 2).

**Table 2:** HIV knowledge and institutional characteristics of healthcare providers, Southwest Ethiopia, 2011(n = 255)

**Variable**	**No (%)**
Training on topics related to stigma and discrimination	
None	157 (61.60)
Once or more	98 (38.40)
Basic HIV knowledge	
High basic HIV knowledge	68 (26.70)
Low basic HIV knowledge	187 (73.32)
In-depth HIV knowledge	
Had in-depth HIV knowledge	99 (38.80)
No in-depth HIV knowledge	156 (61.20)
HIV case load in the last 6 months	
10 or more clients	69 (27.10)
9 or fewer clients	186 (72.90)
Type of healthcare facility	
Hospital	61 (23.90)
Health center	194 (76.10)
Presence of ART service in the healthcare facility	
Present	151 (59.20)
Absent	104 (40.80)

### Perceived institutional support

The mean score was highest for supply-related perceived institutional support (%SM =78.9) and lowest for policy-related institutional support (%SM = 42.3). The scores were reported in both raw mean scores and in the mean scores as the percentage of maximum possible scale score (%SM) (Table 3).

**Table 3:** Mean scores for the perceived institutional support amongst healthcare providers, Jimma zone, Southwest Ethiopia, 2011 (n = 255)

**Emerged factors (scales)**	**Mean raw score ± SD**	**%SM**^**4**^
Supply-related institutional support	12.89 ± 3.17	78.90
Policy-related institutional support	5.54 ± 2.17	42.33
Protocol-related institutional support	16.48 ± 4.41	67.71

^**4**^ (%SM) is the Standardized score as the percentage of possible maximum scale score, and it lies between 0 and 100.

According to the key informants from the health facilities, there were no policies specifically dealing with PLHIV and their healthcare providers. In addition, the respondents reported that no training specifically dealing with stigma and discrimination against PLHIV has been provided to healthcare providers and anti-discrimination policy is non-existent in the healthcare institutions. However, they admitted that the issue of stigma and discrimination was incorporated into the comprehensive HIV training.

The key informants said that HIV-related protocols were availed only, to those healthcare providers who have taken the respective trainings. Regarding supply-related institutional support, shortages of laboratory reagents and drugs were emphasized by the representatives of associations of PLHIV and by FGD participants. Furthermore, FGD participants blamed healthcare providers for attributing the poor care and support services to the shortage of logistics, supplies and budgets.

### Stigma and discrimination against PLHIV

Stigmatization was highest for extra precaution factor (%SM = 66.44) followed by the fear of work-related HIV transmission (%SM = 53.33) and the lack of feelings of safety (%SM = 49.38) (Table 4).

**Table 4:** Mean scores for stigma and discrimination amongst healthcare providers, Jimma zone, Southwest Ethiopia, 2011(n = 255)

**Emerged factors (scales)**	**Mean raw score ± SD**	**% SM**^**4**^
Lack of feelings of safety	19.85 ± 6.88	49.38
Discomfort around PLHIV	10.16 ± 3.94	34.40
The fear of work-related HIV transmission	5.14 ± 1.95	52.33
Value-driven stigma	15.20 ± 6.36	39.04
Unethical handling of PLHIV	8.49 ± 3.80	37.42
Extra precaution	8.99 ± 2.92	66.55
Disclosure(n = 206)	9.66 ± 5.35	31.07

^**4**^ (%SM) is the Standardized score as the percentage of possible maximum scale score, and it lies between 0 and 100.

The existence of frequent discriminatory actions against PLHIV amongst healthcare providers was also implied in the FGDs. Participants stated that discrimination of PLHIV by healthcare providers was “*a frightening issue*”. Discriminatory actions mentioned in the discussions include: unnecessary referral of, and designating of PLHIV, testing and disclosure of the test results without getting informed consents and refusal to treat HIV clients.

The representatives of PLHIV explained that conflicts between healthcare providers and PLHIV arise when healthcare providers unnecessarily refer clients to other facilities. On the other hand, key informants from health centers noted that healthcare providers refer PLHIV only when there are shortages of drugs and reagents and for further management in better equipped hospitals.

FGD participants also mentioned the existence of unofficial disclosure of HIV sero-status of clients. One 31-year old female FGD participant said, *“If a healthcare provider knows that a client is on ART in a different health institution he/she usually refuses to provide services the client is looking for.”*

Representatives of PLHIV have also stated that some PLHIV complain of their sero-status being revealed to others without their knowledge. On the other hand, managers of the health centers explained that unofficial disclosure by the healthcare providers is meant to *“facilitate the health services delivery to PLHIV and ensure that healthcare providers take extra precautions while dealing with HIV-positive clients”.* However, one health center manager commented, *“Introduction of ART coupled with the orientation of the healthcare providers about the importance of confidentiality has reduced the frequency of breaches of confidentiality.”*

The interaction between healthcare providers and PLHIV was also one of the themes emerged from the qualitative data. Both key informants and FGD participants appreciated the good interactions between PLHIV and healthcare providers who work in ART services points. The health managers of the health centers attributed this to the fact that healthcare providers working in the ART services points are managing all matters related to the care to PLHIV.

However, unfavorable relationship was reported by the FGD participants in the case of those healthcare providers working in section other than the ART service points. Expressions to support this claim include: *“xiiqiidhaan nu gaariffatu”* and *“du’a keenya jaallatu*” Afan Oromo meaning: *“it is like we are enemies”* and *“they expectour deaths”, respectively*. FGD participants also blamed healthcare providers for failing to help them to be enrolled in care and support activities related to HIV.

The other theme identified was designation of PLHIV by the healthcare providers. A 43-year old female FGD participant said, *“I heard a healthcare provider saying ‘Pooz nech’ while pointing to somebody”*. ‘*Pooz nech*’is an Amharic phrase meaning “*she is positive”*. Moreover, participants said that healthcare providers designate them saying, “*jarittin maatiihin qabnedhufte*” Afan Oromo, meaning “*those who do not have families are coming*”.

### Predictors of stigma and discrimination

#### The fear-driven stigma

The significant predictors of stigma and discrimination as demonstrated by the lack of feelings of safety were: the lack of in-depth HIV knowledge (p < 0.01), low basic HIV knowledge, low HIV case load (p < 0.01), the lower perception of protocol-related institutional support (p < 0.01), the lower perception of policy-related institutional support (p < 0.05) and the lower perception of supply-related institutional support (p < 0.05). Those healthcare providers with high basic HIV knowledge had an average of 0.29 units lower stigma scores when compared to those healthcare providers with low basic HIV knowledge. In addition, healthcare providers who had high HIV case load had an average of 0.15 units lower stigma scores when compared to those healthcare providers who had low HIV case loads. A unit increment in the perception of policy-related institutional support and a unit increment in the perception of protocol-related institutional support also decreased the stigma score measured by the lack of feelings of safety by an average of 0.13 and 0.12 units respectively (Table 5).

**Table 5:** Predictors of stigma and discrimination measured by fear-driven stigma and unofficial disclosure dimensions, Jimma zone, Southwest Ethiopia, 2011(n = 255)

**Predictors**	**Lack of feelings of safety**			**Discomfort around PLHIV**			**Unofficial Disclosure**		
	**Std ß**	**95%CI for B**		**Std ß**	**95%CI for B**		**Std ß**	**95%CI for ß**	
		**Lower bound**	**Upper bound**		**Lower bound**	**Upper bound**		**Lower bound**	**Upper bound**
Basic HIV knowledge	−0.76^**^	−0.98	−0.54	®NI			® NI		
In-depth HIV knowledge	−0.12^*^	−0.49	−0.01	−0.45	−0.69	−0.22	®NI		
Protocol	−0.12^*^	−0.24	−0.00	−0.11	−0.24	0.02	−0.15^*^	−0.28	−0.01
Supply	−0.11	−0.22	0.01	−0.26^*^*	−0.38	−0.13	®NI		
Policy	−0.13^*^	−0.24	−0.02	®NI			®NI		
High HIV Caseload	−0.15*	−0.61	−0.08	®NI			®NI		
^i^Degree and above	®NI			−0.15^*^	−0.58	−0.08	®NI		
Training	®NI			−0.05	−0.35	0.15	−0.17^**^	−0.63	−0.08
^+^Income 2250 and higher	0.13*	0.05	0.57	®NI			®NI		
Very religious	®NI			0.03	−0.22	0.38	0.20^**^	0.15	0.81
Somewhat religious	®NI			−0.06	−0.42	0.15	0.27^**^	0.30	0.91
ART service present	®NI			−0.06	−0.39	0.13	®NI		
Adj R^2^	0.217			0.125			0.123		

^*^ significant at 0.05 level ** significant at 0.01 level ^i^The comparative group was diploma and certificate.

^+^ The baseline was income of 1233 Eth. Birr or less ®NI: not included in the final model.

NB: Negative values of std β (standardized beta) show that the corresponding factors are the negative predictors for stigma and discrimination, whereas the positive values indicate that the factors are positive predictors.

On the other hand, the lower perception of supply-related institutional support (p < 0.01), and educational status of diploma and certificate (p < 0.01) were significant predictors of discomfort around PLHIV. On average, a unit increment in the perception of supply-related institutional support reduced stigma score measured by discomfort around PLHIV by 0.26 units. Healthcare providers with first degree and higher educational qualifications had an average of 0.15 units lower stigma scores when compared to those with diploma and certificate level educational qualifications (Table 5).

### Unofficial disclosure dimension

Claiming oneself as somewhat religious (p < 0.01), claiming oneself as very religious (p < 0.01), the lack of training on topics related to stigma and discrimination (p < 0.01) and the lower perception of protocol-related institutional support (p < 0.05) were significant predictors of stigma and discrimination measured by unofficial disclosure. On average, the stigma scores of healthcare providers who claimed to be somewhat religious were 0.28 units higher when compared to the scores of those healthcare providers who claimed to be non-religious. In addition, the stigma scores of healthcare providers who claimed themselves as very religious were 0.20 units higher when compared to the stigma scores of those healthcare providers who claimed to be non-religious. Those healthcare providers who had attended training on topics related to stigma and discrimination had an average of 0.17 lower stigma scores when compared to those healthcare providers who had not attended the training. A unit increment in the perception of protocol-related institutional support reduced the stigma score measured by unofficial disclosure, by 0.15 units (Table 5).

#### Value-driven stigma

The lack of in-depth HIV knowledge (p < 0.01), the lower perception of supply-related institutional support (p < 0.01) and the lower perception of protocol-related institutional support (p < 0.05) were significant predictors of value-driven stigma. A unit increment in the perception of supply-related institutional support and a unit increment in the perception of protocol-related institutional support reduced stigma and discrimination measured by value-driven stigma by an average of 0.18 and 0.15 respectively. Healthcare providers having in-depth HIV knowledge had an average of 0.16 units lower stigma scores when compared to healthcare providers who did not have in-depth HIV knowledge (Table 6).

**Table 6:** Predictors of stigma and discrimination measured by value-driven stigma and discrimination dimensions, Jimma zone, Southwest Ethiopia, 2011(n = 255)

**Predictors**	**Value-driven stigma**			**Unethical treatment**			**Extra precaution**		
	**Std ß**	**95%CI for ß**		**Std ß**	**95%CI for ß**		**Std ß**	**95%CI for ß**	
		**Lower bound**	**Upper bound**		**Lower bound**	**Upper bound**		**Lower bound**	**Upper bound**
In-depth knowledge	−0.16*	−0.57	−0.07	0.05	−0.16	0.35	®NI		
Basic knowledge	−0.10	−0.49	0.06	−0.003	−0.28	0.27	®NI		
Protocol	−0.15^*^	−0.27	−0.031	−0.18^**^	−0.30	−0.06	®NI		
Supply	−0.18^**^	−0.30	−0.06	−0.05	−0.17	0.07	®NI		
^iii^Very religious	0.12	−0.004	0.55	0.28^**^	0.39	0.95	®NI		
^iv^Income 1234–2249 Birr	®NI			®NI			−0.22^**^	−0.78	−0.16
^iv^Income 2250 Birr or more	®NI			®NI			−0.07	−0.67	0.31
^i^ Degree and above	®NI			®NI			−0.13	−0.67	0.14
Experience in years	®NI			−0.12^*^	−0.40	0.000	®NI		
**Adj-R**^**2**^	0.110			0.114			0.061		

^*^ Significant at 0.05 level ^**^ significant at 0.01 level ^iii^ The reference group was non religious ^i^ the reference category was diploma and certificate iv the reference category was monthly income less than or equal to 1233 Eth. Birr. ®NI: not included in the final model.

NB: Negative values of std β show that the corresponding factors are the negative predictors for stigma and discrimination, whereas the positive values indicate that the factors are positive predictors.

On the other hand, in the extra precaution scale, healthcare providers with monthly income of 1234–2249 Ethiopian Birr reported 0.20 units lower stigma scores when compared to those healthcare providers with monthly income of 1233 Ethiopian Birr and lower (p < 0.01) (1 US dollar = 17.02 Ethiopian Birr during the data collection period). Healthcare providers with degree and above educational level had an average of 0.17 units lower stigma scores when compared to those healthcare providers having diploma and certificate qualification (Table 6).

#### Discrimination dimension

Claiming oneself to be very religious (p < 0.01), the lower perception of protocol-related institutional support (p < 0.01) and lower years of work experience (p < 0.05) were significant predictors of stigma and discrimination measured by unethical treatment of PLHIV. On average, healthcare providers who claimed to be very religious had 0.28 units higher stigma scores when compared to those healthcare providers who claimed to be non-religious (p < 0.01). A unit increment in the perception of protocol-related institutional support reduced stigma scores measured by unethical treatment of PLHIV, by an average of 0.18 units. A one year increase in work experience also reduced the stigma scores measured by this scale, by an average of 0.12 units (Table 6).

## Discussions

Stigma and discrimination have been obstacles to care and support services in the context of HIV/AIDS. In the current study, stigmatization was highest for the extra precaution scale followed by the fear of work-related HIV transmission (%SM = 52.3). These scores were above the standardized mean (50), indicating some evidences of stigmatization and discrimination.

Healthcare providers who had high basic HIV knowledge had lower stigma scores when compared to those healthcare providers with low basic HIV knowledge. In addition, healthcare providers having in-depth HIV knowledge had lower scores as compared to those healthcare providers who did not have in-depth HIV knowledge. These findings are in agreement with the findings of other studies done elsewhere [5,18,26,48,54-58].

Similarly, healthcare providers who had attended training on topics related to stigma and discrimination had lower stigma scores when compared to those healthcare providers who had not attended the trainings. This finding is in agreement with previous studies, which indicated that formal HIV/AIDS training is significantly associated with less stigmatization and discrimination [5,14,48,55,57,59].

When stigma scores were compared by educational level categories, those healthcare providers with first degree and higher educational level had lower stigma scores when compared to those healthcare providers with diploma and lower educational level. This finding is supported by the findings of Tanzania Stigma Field Test Group; by the study conducted by the USAID/Health Policy Initiative, Task Order 1; and by the study conducted in Bangladesh [25,48,57].

But it is not in agreement the findings of Vyas et al and Li et al, which indicated that medical professionals with more years of education are more likely to discriminate against PLHIV [20,60].

Those healthcare providers with high HIV case load had lower stigma scores when compared to those healthcare providers with low HIV case load. This finding is in agreement with the study done in Barbados and with the study conducted by USAID/Health Policy Initiative, Task Order 1 [48,54].

In our study, a unit increment in the perception of policy-related institutional support had reduced the lack of feelings of safety by an average of 0.13 units. The perception of policy-related institutional support was also negatively correlated with the fear of work-related HIV transmission (r = −0.14 p < 0.05). These findings are supported by the study of Andrewin and Chien [59].

The lack of specific policies or clear guidance related to the care of clients with HIV reinforces discriminatory behaviour amongst healthcare providers [40]. Even though Ethiopia has laws and regulations that protect PLHIV against discrimination [44], in the current study, the perception of policy-related institutional support was low. Key-informants from health centers also stated that there was no special policy that protects PLHIV against discrimination. The key informants also said that there was no special support for healthcare providers working with PLHIV. In addition, all of them denied the existence of anti-discrimination policy and a separate training related to stigma and discrimination for healthcare providers. This underscores the need to focus on clearly communicating anti-stigma and anti-discrimination regulations to healthcare providers and the need for enacting them.

In our study, the perception of supply-related institutional support significantly reduced stigma scores. In addition, in the qualitative part of our study, the shortage of materials and supplies was pointed as the cause of conflicts between PLHIV and healthcare providers. Other studies also showed that the lack of protective and treatment materials favor discriminatory practices and attitudes [14,16]. In the study by Sadow et al, significantly, more healthcare providers were willing to give an injection or set up an infusion if gloves were worn than if there were no gloves [17]. Moreover, the findings of our study are in agreement with the study conducted in China, which showed that the more institutional support healthcare providers were perceived to have, the less discrimination intent they would exhibit against PLHIV [55].

The study by Reis et al indicated that healthcare providers who reported working in facilities that did not always practice universal precautions against HIV transmission were more likely to favor restrictive policies towards PLHIV [16]. In our study, key informants from health centers said HIV-related protocols (including precaution protocol) are available only to those healthcare providers who had taken the respective trainings. The copies of these protocols had not been availed to each healthcare provider except that they find them with their efforts. This can create a gap in healthcare practices. Furthermore, the lower perception of protocol-related institutional support was a significant predictor of unethical treatment of PLHIV, value-driven stigma, unofficial disclosure and the lack of feelings of safety. Therefore, availing these protocols can also contribute to the reduction of stigma and discrimination against PLHIV through the increment of the perception of institutional support.

In disclosure dimension of stigma and discrimination, the healthcare providers who claimed to be very religious had significantly higher stigma scores when compared to those healthcare providers who claimed to be non-religious (p < 0.01). And, those healthcare providers who claimed to be somewhat religious had significantly higher stigma scores when compared to those healthcare providers who claimed to be non-religious. The stigma score measured by unethical treatment scale also varied with perceived religiousness. The healthcare providers who claimed to be very religious had higher stigma scores when compared to those healthcare providers who claimed themselves as non-religious (p < 0.01). These findings are in agreement with the findings of Andrewin and Chien and with the study conducted in Bangladesh [57,60]. This indicates that healthcare providers share not only stigmatizing attitude related to their occupation, but also the stigmatizing attitude present in their communities.

It was noted that healthcare providers unofficially disclose the HIV sero-status of clients in order to facilitate the healthcare given to the clients and to *“ensure that healthcare providers take extra precautions while dealing with HIV-positive clients”*. Similar claims were made to justify unofficial discloser of sero-status by healthcare providers in an earlier study from India [18].

Designation of some phrases to PLHIV was also reported to be common amongst the healthcare providers in the study area. Besides, healthcare providers have failed to involve PLHIV in the care and support activities related to HIV. Involvement of PLHIV in these activities has been reported improve empowerment of the PLHIV and the probability of contact with healthcare providers. This contributes to the reduction of negative attitudes towards PLHIV [48,54].

In the current study, both method and person triangulations were employed, which has increased the credibility and richness of the findings. However, it has to be noted that the findings of this study mainly reflect situation in the district healthcare settings (district hospitals and health centers) of Ethiopia. Therefore, the findings should be interpreted with caution. Replicability of the findings should be checked through further study at different levels of the health system. The responses might have been liable to social desirability bias, which might under estimate the level of stigma and discrimination. However, the use of self-administered questionnaire and replacement of names of the healthcare providers with codes were both helpful to minimize this problem.

## Conclusion

Our study indicated that equipping healthcare providers with knowledge on HIV, through the provision of protocols and trainings, is of paramount importance in reducing stigma and discrimination against PLHIV amongst healthcare providers. The healthcare providers’ awareness of anti-stigma and anti-discrimination rules and regulations also contributes to the reduction of stigma and discrimination. Nevertheless, in our study, only few proportions of healthcare providers had in-depth HIV knowledge. Considerable proportion of the healthcare providers have attended trainings specifically focusing on stigma and discrimination. The perception of policy-related institutional support was also low. In addition, HIV-related protocols were not availed to each healthcare provider.

Healthcare providers share not only stigma and discrimination related to their occupation, but also stigma and discrimination present in their communities. This implies that community-based anti-stigma and anti-discrimination interventions may contribute to the reduction of stigma and discrimination amongst healthcare providers.

Taking into account what have already been outlined in this report, we would like to forward the following recommendations to all concerned bodies. The Federal Ministry of Health, the regional bureaus, zonal health departments, district health offices and healthcare institutions should avail protocols related to HIV to each healthcare provider in healthcare settings. They should orient healthcare providers about the contents and relevance of the HIV-related policies. In addition, they should provide the opportunity for trainings on stigma and discrimination to healthcare providers from time to time. Organizations working on HIV/AIDS should extend HIV-related care and support services and anti-discrimination interventions to districts. And they should involve PLHIV and religious leaders in these activities. Healthcare providers should strive to update their knowledge on HIV/AIDS. Further study should be conducted in different levels of healthcare settings.

## Competing interests

The authors declare that they have no competing interests.

## Authors’ contributions

GTF was involved in the design of the study, data analysis, and interpretation of the findings, report writing and manuscript preparation. LA was involved in the design of the study, data analysis and review of the report. EG was involved in the design of the study, analysis and interpretation of the data, and review of the report. MW was involved in the design of the study, analysis and interpretation of the data, and writing and review of the report and manuscript. All the authors have read and agreed on the submission of the final manuscript.

## Pre-publication history

The pre-publication history for this paper can be accessed here:

http://www.biomedcentral.com/1471-2458/12/522/prepub
